# Micron-Sized Fe_3_O_4_/PCL Biocomposite Scaffolds to Attract Magnetic Nanoparticles for Targeted Drug Delivery

**DOI:** 10.3390/bioengineering12040371

**Published:** 2025-04-01

**Authors:** Jianhua Ge, Riley Drees, Aoran Wang, Bo Zhu, Shang-You Yang

**Affiliations:** 1 Key Laboratory for Liquid-Solid Structural Evolution & Processing of Materials, Shandong University, Jinan 250061, China13605317708@139.com (B.Z.); 2 Department of Biological Sciences, Wichita State University, Wichita, KS 67260, USA; rgdrees@shockers.wichita.edu; 3 School of Materials Science & Engineering, Shandong University, Jinan 250061, China; 4 Department of Orthopaedic Surgery, University of Kansas School of Medicine-Wichita, Wichita, KS 67214, USA

**Keywords:** magnetic scaffold, magnetic drug delivery, nanoparticles trafficking, biocompatibility, mouse model

## Abstract

Adjuvant chemotherapy is a critical regime in cancer treatment. The magnetic targeted drug delivery system (MTDDS) can selectively aggregate chemotherapy agents at the target areas, which has attracted great attention due to its safety, high efficiency, and minimal side effects on the human body. It would be ideal to establish a tissue engineering scaffold that can not only reconstruct the defect from the surgical tumor removal, but also serve as a magnetic station to attract MTDDS to the local site to enhance the targeted drug delivery. The current study constructed polycaprolactone magnetic tissue engineering scaffolds with various micrometer-sized magnets. The degradation properties of the scaffolds were assessed in simulated body fluid (SBF), and primary mouse bone marrow stromal cells were used to evaluate the biocompatibility of the scaffolds to osteoblast differentiations. The scaffolds were further examined by implantation to an air pouch model on the back of BALB/c mice. The in vitro data suggested that up to 40% of micron-sized magnetite can be used to formulate porous polycaprolactone (PCL) scaffolds with comparable biocompatibility to the PCL-alone scaffold. A mouse study revealed that the intro-peritoneal injected fluorescence-magnetic particles were collectedly enriched in the mouse air pouch tissues containing the 20% magnetic/PCL scaffolds. Histological assessment and the real-time PCR results of the air pouches confirmed the benign biocompatibility of the implanted magnetic scaffolds.

## 1. Introduction

Solid cancer is one of the leading causes of mortality, and effective treatment of cancer is very challenging. The efficacy of conventional chemotherapy is reduced due to the non-specific distribution, low efficiency, and significant toxicity of many chemo agents in the human body when administered at high doses [1,2,3,4]. Cancer patients often require chemotherapy after surgical resection of the lesion. Chemotherapy drugs are toxic, and once they enter the human body, they will spread throughout the body, making it difficult to concentrate on the lesion. Therefore, chemotherapy can bring systemic toxicity to patients [5,6,7,8,9]. Meanwhile, it is necessary to maintain a higher dosage of chemotherapy drugs for systemic administration in order to achieve the therapeutic concentration at the tumor site, which further enhances the toxicity of drugs to adjacent and remote normal tissues. The concept of a drug delivery system (DDS) has hence been popularized. Among them, the magnetic targeted drug delivery system (MTDDS) has attracted close attention due to its safety, high efficiency, and minimal side effects on the human body [10,11,12,13]. However, most research on magnetic targeted drug delivery systems relies on the strategy of using external magnetic fields to guide magnetic drugs to the lesion [11,14,15,16,17,18]. Although this may be relatively straightforward for the treatment of surface lesions or superficial organs, the technique appears difficult and inconvenient for the accurate delivery of MTDDS to deeper tissues and organs such as bones.

In the clinical management of malignant tumors such as osteosarcoma, a common practice is the surgical removal of the primary tumor with adjuvant chemotherapy before and/or after surgery. Since the scaffolds or man-made devices are inevitably implanted after tumor resection to replace the void, the research concept of this study is that by implanting a magnetic tissue engineering scaffold into the void space left by the surgical resection, it not only serves as a tissue reconstruction scaffold but also become an attraction source to gather/concentrate the chemotherapeutic agent-capsulated magnetic particles for targeted drug delivery. Previous studies from our lab and others have fabricated and evaluated various drug-containing magnetic nanoparticles [19,20,21]. Recent focus, however, has been directed at constructing magnetic tissue engineering scaffolds and studying their properties in vitro and in vivo.

In the current study, we prepared ferroferric oxide (Fe_3_O_4_)/polycaprolactone (PCL) magnetic tissue engineering scaffolds and tested their degradation performance in SBF. PCL is an FDA-approved biomaterial for sutures and implantable medical device practice due to its benign biocompatibility and biodegradability [22]. In mixing appropriate concentrations of Fe_3_O_4_, sufficient magnetic attraction power and biocompatibility were assessed. This was followed by an in vitro evaluation of the cytotoxicity/biocompatibility of the scaffolds to the osteoblasts induced by BALB/c mouse bone marrow stem cells. Lastly, a murine air pouch model was adopted to examine the feasibility of MTDDS trafficking and tissue responses to the particles in the pre-clinical setting.

## 2. Materials and Methods

### 2.1. Fe_3_O_4_/PCL Scaffolds Preparation

Polycaprolactone (PCL) scaffolds with iron(II,III) oxide (Fe_3_O_4_) were prepared using a sodium chloride (NaCl) particulate leaching technique [23]. In brief, PCL (Mn80,000, Cat# 440744, MilliporeSigma) was dissolved in tetrahydrofuran (Cat# 34865, MilliporeSigma) at 40 °C for 12 h, followed by homogeneously mixed with Fe_3_O_4_ particles (<5 μm, 95%, Cat# 310069, MilliporeSigma, St. Louis, MO, USA) at 0:1 (E0), 0.05:0.95 (E1), 0.10:0.90 (E2), 0.20:0.80 (E3) and 0.40:0.60 (E4) *wt/wt* ratios (Table 1). NaCl particles (Cat# S9888, MilliporeSigma) sized in 355–500 um were also added to the mixture at 9-fold of the total weight of Fe_3_O_4_ and PCL to generate a controlled porosity in the scaffold. The mixtures were stirred to a viscous slurry prior to casting into a mold. After evaporation of the solvent, the samples were taken out of the mold and washed in excess distilled water to leach out NaCl. The ultrastructure of scaffolds was observed using a scanning electronic microscope (SEM, Hitachi SU-70, Hitachi High-Tech America, Inc., Dallas, TX, USA).

### 2.2. Degradability of Scaffolds in Simulated Body Fluid

The simulated body fluid (SBF) used in this research was prepared according to the literature [24] and has a pH value of 7.4. Its components are similar to human plasma. Scaffold dice (10 mm × 10 mm × 2.5 mm) were incubated in 5 mL SBF in test tubes, which were agitated at 37 °C at a rate of 160 rpm. The SBF solution was changed weekly.

The dry weight of scaffolds was measured before being immersed in the SBF (designated as “Wi”). After incubation in the SBF for one week, scaffolds were taken out and washed with ddH_2_O thoroughly. After being dried out, their weight was measured again (designated as “Ws”). The percentage of mass loss was recorded as mass loss (%) = (Wi − Ws)/Wi × 100%. If Wi < Ws, the calculation formula would be changed to (Ws − Wi)/Wi × 100% as the increased weight ratio of the scaffolds shown in the figure. pH value change of the SBF and weight change of scaffolds were continually recorded for 4 weeks.

### 2.3. Mouse Cells Culture

Primary bone marrow-derived stromal cells (BMSCs) were isolated from Balb/C mice of 6–8 weeks of age (The Jackson Laboratory, Bar Harbor, ME, USA) and were induced to differentiate to osteoblasts in DMEM media supplemented with 10% fetal bovine serum (FBS, heat-inactivated, ThermoFisher Scientific, Carlsbad, CA, USA), 10 mM β-glycerolphosphate (Cat# G9422, MilliporeSigma), 100 mM L-ascorbic acid (Cat# 4544, MilliporeSigma), and 10 nM dexamethasone (Cat# D4902, MilliporeSigma), 2 mM glutamine (Cat# 25030-081, ThermoFisher Scientific), and 100 U/mL penicillin-Streptomycin (Cat# 15140-122, ThermoFisher Scientific) [25]. The medium was changed every third day until it was used for the scaffold cytotoxicity experiments.

### 2.4. Cytotoxicity Assay of Fe_3_O_4_/PCL Scaffolds

Each sample (10 mm × 10 mm × 2.5 mm dice) was immersed in 1 mL culture medium in a sterile tube for 24 h at 37 °C before harvesting the supernatant for Day 1 release. The same amount (1 mL) of fresh medium was added back to the tube. The medium elution harvest was repeated daily until Day 7 release media were yielded.

Primary mouse osteoblasts were seeded in a 96-well plate at 10^4^/100 μL medium/well for 24 h in an incubator (37 °C, 5% CO_2_ in air) before the introduction of the samples release media (100 μL medium/well), including control wells with fresh medium. AlamarBlue^®^ reagents (Cat# Y00-025, ThermoFisher Scientific) at 1 to 10 ratios were added to the culture medium. Absorbance detection of the culture media was performed 6 h later on a spectrophotometer (SpectraMax Plus384, Molecular Devices, LLC., San Jose, CA, USA) after transferring to a new reading plate [26]. Cells were continued to culture in a fresh medium and repeated the above AlamarBlue assays each day for a total of 7 days. The cell proliferation/cytotoxicity ratios among groups were calculated based on the absorbance readings of alamarBlue^®^ at 570 nm and normalized with the 600 nm values.

### 2.5. Animal Experiment

All the animal procedures were approved by the Institutional Animal Care and Use Committee. Twenty Balb/C mice of ~20 gm body weight (The Jackson Laboratory) were recruited to establish the air pouch model as previously described [27]. Briefly, 3 mL of filtered air was subcutaneously injected into the back of the mice, with a repeated 1 mL of air injection 3 days later. At day 7, when the air pouches were established, scaffold dices (8 mm × 8 mm × 1 mm) of E0 and E3 were surgically implanted into the pouches. On the following day, 0.5 mL fluorescence-magnetic suspensoids were peritoneally injected into each mouse. The mice were sacrificed 5 days later. The pouches were collected for histological and molecular assessments.

The fluorescence-magnetic suspensoids were fabricated by mixing 14 mg of Fe_3_O_4_ particles (<50 nm, MilliporeSigma) with 4 mg 1,6-Diphenyl-1,3,5-hexatriene (DPH, 98%, Cat# D208000, MilliporeSigma) in 10 mL of sterile PBS.

### 2.6. Histological Assessment

Cryosections of implanted scaffolds at 10 μm thickness were processed at the Pathology Core in Via Christi St. Francis Hospital and stored at −20 °C till used. Hematoxylin and eosin (H&E) staining was performed, and the images of stained sections were digitally captured under a Nikon Eclipse fluorescent microscope (Nikon, Melville, NY, USA). The fluorescence images of the frozen sections of the pouches and organ tissues were digitally captured under a dark-field fluorescence Microscope (Axio Imager A2, Carl Zeiss Microscopy, LLC., White Plains, NY, USA).

### 2.7. Real-Time PCR

The expression of inflammatory cytokines in the air pouch tissues around the implant scaffolds was quantified by a real-time polymerase chain reaction (RT-PCR) technique with the standardized protocol previously described [28,29]. In brief, mouse pouch tissues were homogenized using a Polytron Homogenizer (Brinkmann Instruments, Riverview, FL, USA), and total RNAs from the homogenates were isolated by TRIzol^TM^ (Cat# 15596-026, ThermoFisher Scientific)/chloroform (Cas# 67-66-3, MilliporeSigma). Reverse transcription and real-time PCR were performed in the StepOne Plus, Real-Time PCR System (Applied Biosystems, ThermoFisher Scientific), with murine IL-1β, TNF-α and IL-6 primer pairs. The fluorescent signals were recorded dynamically. Normalization and analysis of the reporter signal (∆Rn) at the threshold cycle were carried out, and the relative target gene quantitation among samples was calculated using the software provided by the manufacturer [30].

### 2.8. Statistical Analysis

In vitro experimental data from 3 individual experiments were combined for statistical analysis. The sample size of the animal experiment was estimated using a PS^TM^ program (Power and Sample Size Calculations, version 3.1.2). A student T-test and one-way analysis of variance (ANOVA) with LSD post hoc multiple sample comparisons were performed among groups (IBM SPSS, version 22). Data were expressed as the mean, standard error of the mean (SEM), and a *p*-value of less than 0.05 was considered a significant difference.

## 3. Results

### 3.1. Scaffold Morphology Under Scanning Electron Microscope (SEM)

**Figure 1:**
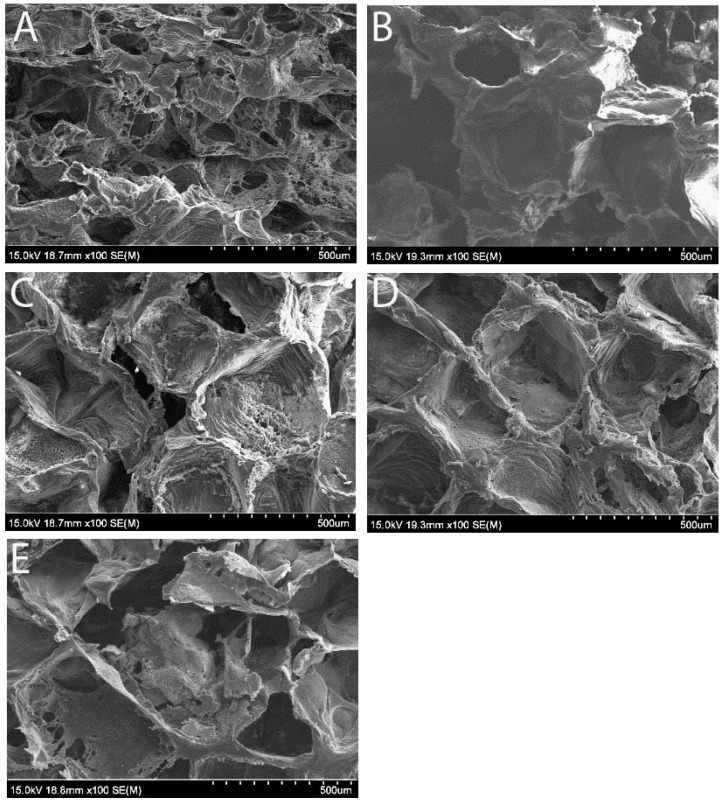
Representative SEM images of the Fe_3_O_4_/PCL scaffolds with various concentrations of micron-sized magnetic particles. (**A**–**E**) illustrate samples containing 0, 5, 10, 20, or 40% of Fe_3_O_4_ particles. The porosity appears uniform after the NaCl leaching process. Scale bars (500 µm/10 ticks) are located at the bottom of each image panel.

**Figure 2:**
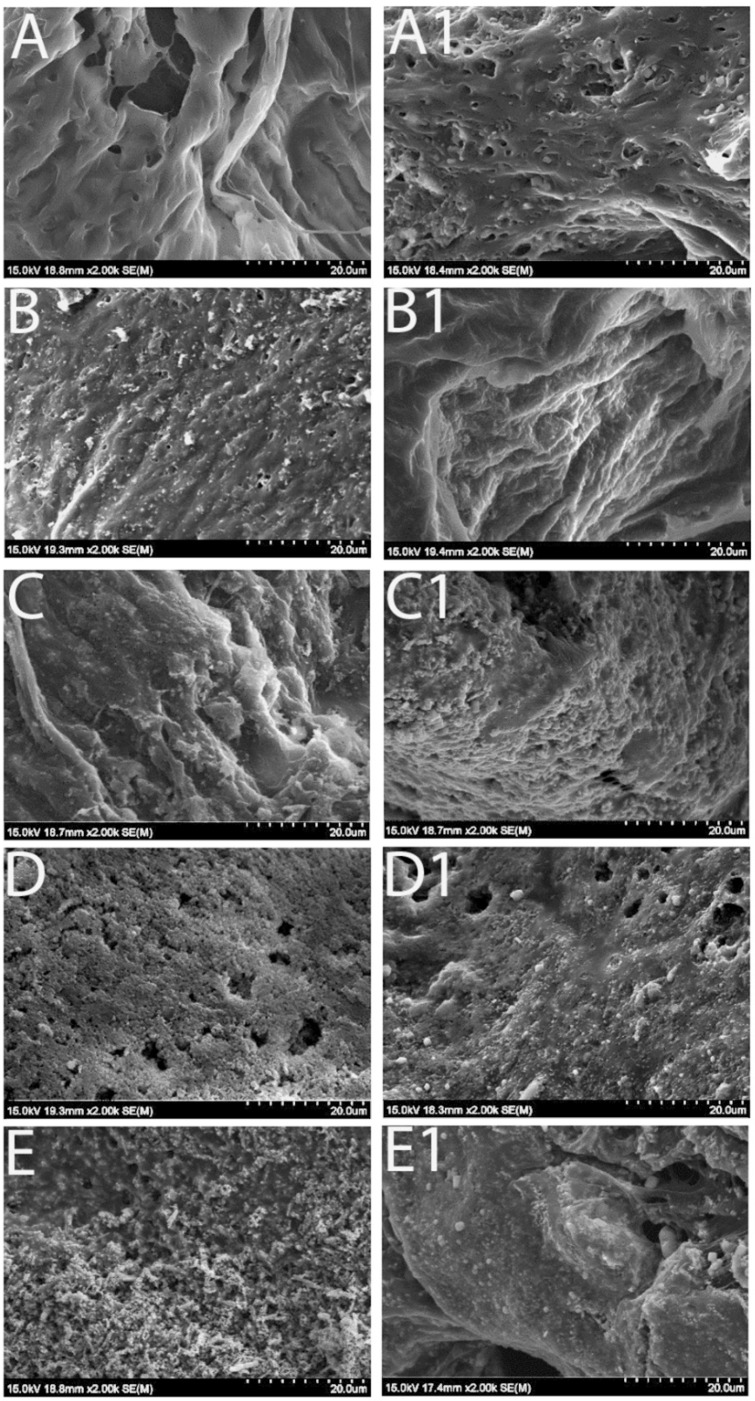
High magnification of SEM images revealing the morphological changes of the scaffolds with various concentrations of the magnetic particles. (**A**–**E**) illustrates samples containing 0, 5, 10, 20, or 40% of Fe_3_O_4_ particles. (**Left column**): prior to immersion in SBF; and (**Right column**): immersion for 2 weeks (scale bars indicate 20 µm/10 ticks at the bottom of the images).

SEM images confirmed that the scaffolds exhibited a uniform open-porous structure on their surface, the same size of porogen (NaCl particles, 355–500 µm) regardless of the Fe_3_O_4_ concentration, which is crucial for cell adhesion and proliferation (Figure 1). Our previous study [31] demonstrated that increasing the Fe_3_O_4_ ratio enhanced the magnetic properties of the scaffolds but reduced their structural stability. When the Fe_3_O_4_ content exceeded 40%, the scaffolds became difficult to fabricate and were prone to fragility. In the current study, we tested five different weight ratios of Fe_3_O_4_/PCL scaffolds, with Fe_3_O_4_ concentrations of 0%, 5%, 10%, 20%, and 40%. Although all scaffolds maintained a consistent open-porous structure, SEM imaging at higher magnification revealed that in the 5% and 10% Fe_3_O_4_ samples (Figure 2B,C), the Fe_3_O_4_ particles were evenly dispersed within the PCL matrix, with a clear separation between the granules. In contrast, the samples with higher Fe_3_O_4_ concentrations (20% and 40%) appeared to be densely packed with particles (Figure 2D,E).

### 3.2. Degradability of Scaffolds in SBF

**Figure 3:**
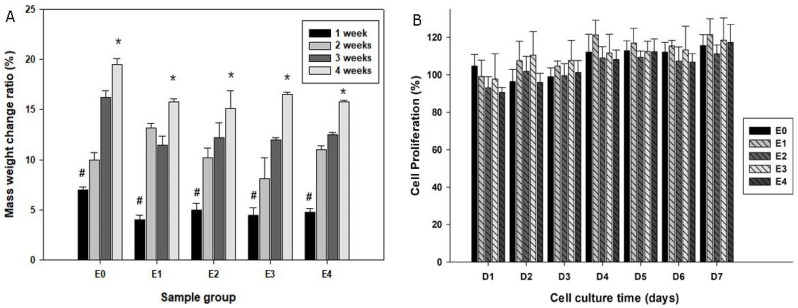
Plot (**A**) quantifies the mass changes of the scaffolds following immersion in SBF over periods of time (* *p* < 0.05 compared to #), while (**B**) summarizes cell growth patterns with co-culturing of elution from various groups of scaffolds by an alamarBlue^®^ proliferation assay.

To test the stability of the Fe_3_O_4_ embedded scaffolds, they were immersed in SBF with physiological pH at 37 °C for a period of time. Figure 2 illustrates the morphological appearance of the samples prior to and after SBF immersion for 2 weeks. Some sediments appeared on the original smooth surface of the pure PCL scaffolds after immersion in SBF (Figure 2(A1)), and the morphological changes were also noticeable on other groups of scaffolds: the original Fe_3_O_4_ particles became smooth but at the same time the new sediments formed under the SEM. Further, dry weight measurements of the scaffolds prior to and after SBF immersion were performed among groups. All the samples gained mass weight following immersion in the simulated body fluid, and the scaffold mass addition was correlated with the immersion time (Figure 3A).

### 3.3. Biocompatibility of the Scaffolds

To test the biocompatibility of the scaffolds, the daily eluted SBF of the scaffolds was added to the primary mouse osteoblast cultures. The alamarBlue^®^ proliferation assay suggested comparable cell proliferation patterns among all groups over the experimental period, suggesting that the components released from the scaffolds were not likely to be cytotoxic or inhibit cell growth (Figure 3B).

### 3.4. Murine Air Pouch Model for Targeted Drug Delivery

**Figure 4:**
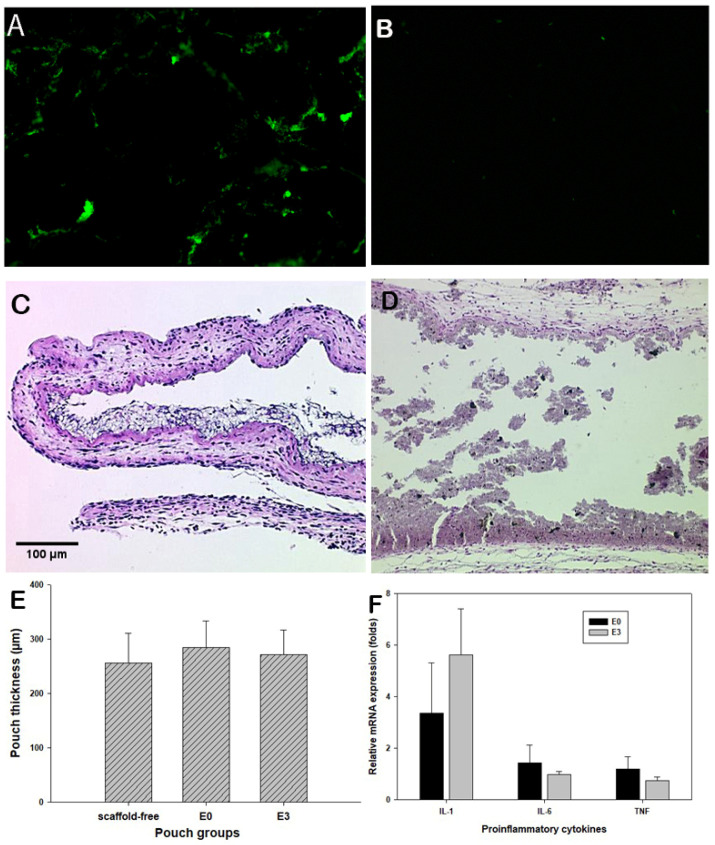
Mouse air pouch model study: Dark-field images of the frozen sectioned air pouches showing fluorescent aggregation in those with 20% Fe_3_O_4_/PCL scaffolds (**A**) but absence in PCL alone pouches (**B**). H&E-stained sections reveal the benign tissue response to E0 or E3 samples (**C**,**D**). Panel (**E**) summarizes the mean air pouch thickness among groups, and (**F**) plots the mRNA expression levels of proinflammatory cytokines in the harvested pouch tissues that interacted with E0 or E3 scaffolds.

To ensure the effective magnetic attraction for potential drug delivery, the trafficking of the fluorescent magnetic particles was examined using the mouse air pouch model [27]. Following peritoneal injection of fluorescent particles, extensive fluorescent signals were accumulated in the pouches with Fe_3_O_4_/PCL scaffolds, while no fluorescence appeared on pouches bearing PCL-alone scaffolds (Figure 4A,B). It is apparent that magnetic composite scaffolds have great potential to attract/home magnetic drug delivery substances. Histological assessment of the pouch tissues bearing Fe_3_O_4_/PCL scaffolds illustrated benign tissue response to the embedded materials, no significant inflammatory cell infiltrations (Figure 4C,D), and inflamed pouch tissues compared to the scaffold-free control group (Figure 4E). Real-time PCR analysis did not reveal significant elevation of IL-1α, IL-6, and TNFβ expressions among the groups (Figure 4F).

## 4. Discussion

MTDDS has attracted widespread attention for its ability to gather chemotherapy drugs at the affected area through magnetic field guidance, thereby reducing the chemo-agents’ systemic side effects. Studies have shown that external magnetic fields may guide magnetic chemotherapy drugs to the affected area for targeted drug delivery [32]. The use of external magnetic fields increases the difficulty of drug administration in clinical practice and may not be sufficient to accurately dispatch drugs to certain targeted areas. We have been investigating an implantable magnetic tissue engineering scaffold to serve as both a tissue void filler following surgical tumor resection and an internal driving force for drug enrichment towards the affected area. Previous in vitro studies from our laboratories have suggested Fe_3_O_4_/poly(lactic-co-glycolic acid) and Fe_3_O_4_/chitosan biocomposites possessed good biocompatibility with high magnetism [31,33]. Mixing various-sized magnet particles with polycaprolactone (PCL) was also examined to screen magnetic composites for their mechanical properties and cell biocompatibility [26]. The current study further evaluated the Fe_3_O_4_/PCL scaffold mass changes during the extended immersion in a simulated body fluid. More importantly, this investigation evaluated, using a murine model, the in vivo biocompatibility and internal magnetic particles’ trafficking in response to variously concentrated Fe_3_O_4_ granules in PCL composites.

Scaffolds used in tissue engineering should remain stable before the implanted cells produce their own extracellular matrix [34], prior to their degradation. There was an interesting finding that the mass weight of Fe_3_O_4_/PCL scaffolds increased during immersion in SBF for 2 weeks or more. Material sediments were deposited on the scaffolds, and the dry weights were increased in an immersion time-dependent fashion. These new sediments appear to be hydroxyapatite, as we reported previously [33]. It is apparent that the deposition rate of sediments to the scaffolds was higher than the degradation rate of the scaffolds. The cell proliferation assay also indicated that the biocomposite scaffolds were stable, and no cytotoxic elution was generated during the testing period. The pH values of the SBF elution samples, up to 4 weeks, did not show significant variations.

To assess the functionality and biocompatibility of the magnetic biocomposite scaffold, a murine air pouch model was adopted. The air pouch model has broadly been used as an in vivo model to investigate localized inflammation, which we have adapted to screen tissue responses to a variety of biomaterials, including their chemical composition, particle sizes, shapes, and concentration. The established air pouches sensitively exhibit inflammatory cellular infiltration and expression of inflammatory mediators depending on the pathophysiological natures of the testing materials. In the current study, established air pouches were surgically implanted with either pure PCL scaffold (E0) or magnetic scaffolds containing 20% Fe_3_O_4_ (E3). The benign tissue response to the two types of scaffolds was confirmed by the histochemical/histological assessment and molecular analysis. More importantly, this study amazingly observed that significantly enhanced fluorescent signals were attracted back into the air pouches containing Fe_3_O_4_/PCL biocomposite scaffolds at 5 days after peritoneal injection of the fluorescent-labeled magnetic particles, indicating the successful particle homing.

## 5. Conclusions

This report suggests that the inclusion of certain concentrations of micron-sized Fe_3_O_4_ in PCL can be fabricated into porous biocomposite scaffolds that are biocompatible, sustainable, and potentially sufficient to attract MTDDS for targeted delivery. Further investigations are warranted to evaluate the therapeutic efficacy of the magnetic engineering scaffolds and potential long-term safety issues using animal models of experimental tumors.

## Figures and Tables

**Table 1:** Composition of Fe_3_O_4_/PCL scaffolds.

Scaffold ID	Fe_3_O_4_ (*w*/*w*, %)	PCL (*w*/*w*, %)
E0	0	100
E1	5	95
E2	10	90
E3	20	80
E4	40	60

## Data Availability

All the research data can be shared by requesting the first and corresponding authors.

## Abbreviations

The following abbreviations are used in this manuscript:

PCL	polycaprolactone
MTDDS	magnetic targeted drug delivery system
SBF	simulated body fluid
H&E	hematoxylin and eosin staining
RT-PCR	reverse transcription-polymerase chain reaction
